# Control of Astrocyte Progenitor Specification, Migration and Maturation by *Nkx6.1* Homeodomain Transcription Factor

**DOI:** 10.1371/journal.pone.0109171

**Published:** 2014-10-06

**Authors:** Xiaofeng Zhao, Yidan Chen, Qiang Zhu, Hao Huang, Peng Teng, Kang Zheng, Xuemei Hu, Binghua Xie, Zunyi Zhang, Maike Sander, Mengsheng Qiu

**Affiliations:** 1 Institute of Developmental and Regenerative Biology, Zhejiang Key Laboratory of Organ Development and Regeneration, College of Life and Environmental Sciences, Hangzhou Normal University, Hangzhou, China; 2 Department of Anatomical Sciences and Neurobiology, School of Medicine, University of Louisville, Louisville, Kentucky, United States of America; 3 Departments of Pediatrics and Cellular and Molecular Medicine, Pediatric Diabetes Research Center, University of California San Diego, La Jolla, California, United States of America; Temple University School of Medicine, United States of America

## Abstract

Although astrocytes are the most abundant cell type in the central nervous system (CNS), little is known about their molecular specification and differentiation. It has previously been reported that transcription factor Nkx6.1 is expressed in neuroepithelial cells that give rise to astrocyte precursors in the ventral spinal cord. In the present study, we systematically investigated the function of *Nkx6.1* in astrocyte development using both conventional and conditional *Nkx6.1* mutant mice. At early postnatal stages, *Nkx6.1* was expressed in a subpopulation of astrocytes in the ventral spinal cord. In the conventional *Nkx6.1KO* spinal cord, the initial specification of astrocyte progenitors was affected by the mutation, and subsequent migration and differentiation were disrupted in newborn mice. In addition, the development of VA2 subtype astrocytes was also inhibited in the white matter. Further studies with *Nkx6.1* conditional mutants revealed significantly delayed differentiation and disorganized arrangement of fibrous astrocytes in the ventral white matter. Together, our studies indicate that *Nkx6.1* plays a vital role in astrocyte specification and differentiation in the ventral spinal cord.

## Introduction

Astrocytes and oligodendrocytes are macroglial cells found in all regions of the central nervous system (CNS). It is estimated that macroglial cells constitute as many as 90% of cells in some regions of the CNS, with astrocytes being the predominant cell type. Although both glial cell types act to support the activities of neurons, oligodendrocytes and astrocytes have clearly distinct functions. The primary function of an oligodendrocyte is to form myelin sheaths around multiple axons for rapid transmission of electrical pulses along axons. In contrast, astrocytes play many diverse roles, both supportive and active, in the functioning of the CNS. Among those are the regulation of ion and neurotransmitter concentrations, formation of the brain blood barrier, modulation of synapse formation and efficacy [1], and induction of neurogenesis in the adult brain [2]. Thus, it is unsurprising that abnormalities in astrocyte density and functioning have recently been implicated in several common neurological diseases including neuropathic pain, depression, and schizophrenia [3], [4].

Spinal cord has served as an excellent model system to study the origin and molecular specification of neurogenesis and gliogenesis due to its relatively simple anatomy and structures. In the developing spinal cord, neuroepithelial cells in the ventricular zone (VZ) first give rise to neurons which subsequently migrate away from the ventricular zone by radial migration. At later stages, neuroepithelial cells switch to produce glial cells, i.e. astrocytes or oligodendrocytes. In the past decade, great progress has been made in our understanding of the origin and molecular control of oligodendrocyte development. During gliogenesis, early oligodendrocyte progenitor cells (OPCs) originate from the ventral motor neuron progenitor domain (pMN) of the ventral neuroepithelium [5], [6], but a small number of OPCs are also generated from dorsal neural progenitor cells at later stage [7], [8]. Recent molecular and genetic evidence suggests that astrocytes are produced from other domains of neural progenitor cells, particularly from the p1-p3 domains in the ventral spinal cord [9], [10]. Although considerable progress has been made in our understanding of the molecular specification of oligodendrocytes, the molecular mechanisms that control the development of astrocytes have been lagging, partly due to the lack of well-defined stage-specific markers for astrocyte lineage.

Previous studies have demonstrated that the *Nkx6.1* homeodomain transcription factor is widely expressed by neural progenitor cells within the ventral neural tube and it plays a prominent role in ventral neural patterning and neurogenesis [11]-[13]. During gliogenesis, *Nkx6.1* expression is retained in neuroepithelial cells in the ventricular zone (VZ), including the progenitor cells that produce astrocyte precursors [14]. More recently, it has been shown that the identity of positionally distinct ventral astrocyte subtypes (VAs) is determined by Nkx6.1 [15]. In this study, we showed that *Nkx6.1* is selectively expressed by ventral astrocytes after they migrate away from the VZ to the surrounding parenchyma. *Nkx6.1* ablation leads to abnormal specification, delayed differentiation and disorganized morphology of ventral astrocytes, indicating an important role for *Nkx6.1* in the development of astrocytes in the ventral spinal cord.

## Materials and Methods

### Animals

Mice used in this study were handled according to the protocols approved by Institutional Animal Care and Use Committee (IACUC), University of Louisville (IACUC: 12034). C57BL/6N mice were obtained from Jackson Laboratory. The *Nkx6.1* homozygous null (*KO*) embryos were obtained by the interbreeding of double heterozygous animals. For generation of tissue-specific *Nkx6.1* conditional mutant mice, *Nkx6.1* conditional knockout *hGFAP^Cre/+^;Nkx6.1^flox/flox^* mice were bred with the *hGFAP^Cre^* line to generate tissuespecific *Nkx6.1*-knockout mice. For mouse genotyping, genomic DNA was extracted from embryonic tissues or mouse tails and subsequently used for genotyping by Southern analysis or PCR. Genotyping protocols for *Nkx6.1KO* mouse line was described in Sander et al. [16], and genotyping protocol for *hGFAP^Cre^* and *Nkx6.1^flox^* mouse lines was described previously [17], [18]. For statistical analyses of cell number and relative expression level of target proteins was calculated from each genotype animals (n = 3).

### *In Situ* RNA Hybridization and immunofluorescence staining

*In situ* hybridization (ISH) was performed according to Schaeren-Wiemers and Gerfin-Moser [19] with minor modifications. Animals were deeply anesthetized and perfused with 4% paraformaldehyde (PFA), and tissues were isolated and postfixed in 4% PFA at 4°C overnight. Fixed spinal cord tissues were embedded in OCT medium and sectioned on a cryostat. Frozen sections (16 µm thick) were subjected to ISH with digoxigenin-labeled riboprobes. Double immunofluorescent procedures were described previously [7]. The dilution ratio of antibodies is as follows: anti-S100B (Millipore Bioscience Research Reagents, 1∶1000), anti-GS (Sigma, 1∶500), anti-BLBP (Abcam, 1∶500), anti-Olig2 (Abcam, 1∶3000), anti-NeuN (Abcam, 1∶100), anti-Iba1 (Wako, 1∶500), anti-Sox10 (1∶3000, kind gift of Dr. Michael Wegner), anti-GFAP (Millipore Bioscience Research Reagents, 1∶500), anti-Nkx6.1 (Developmental Studies Hybridoma Bank, University of Iowa, Iowa City, IA; 1∶50) [20]. For ISH experiments, tissues were hybridized with digoxigenin-labeled *Fgfr3*, *Glast*, *GFAP*, *Reelin* and *Slit1* riboprobes. For double labeling experiments, tissues were first subjected to RNA *in situ* hybridization (ISH) with *Fgfr3* riboprobe, followed by anti-Nkx6.1 immunohistochemical staining with ABC kit as described previously [21].

### Western blotting

Spinal cord tissues were lysed in tissue lysis buffer (Sigma) with protease inhibitor cocktail (Sigma). 30 ng protein from control and mutant tissues was loaded for SDS-PAGE electrophoresis and subsequently detected with anti-Olig2 (Abcam, 1∶10000) anti-GFAP (Millipore Bioscience Research Reagents), anti-Nkx6.1 (DSHB Inc.), and mouse anti-GAPDH (Sigma, 1∶5000) antibodies according to the standard protocol. The optical density of blots on films was assessed with the analysis tool in Quantity One software of BIO-RAD ChemiDoc XRS (Biorad, USA) and the relative densitometric values were used for statistical analyses on the expression level of target proteins.

### Primary Astrocyte culture and Nkx6.1 knockdown in vitro

Cerebral cortices from P0 s.d. rats were dissected out, minced and digested in 0.25% trypsin at 37°C. The digestion was stopped by the addition of Dulbecco's modified Eagle's medium (DMEM)/F12 containing 10% fetal bovine serum (FBS). The dissociated cells were plated in a 75 cm^2^ tissue culture flask coated with 100 µg/ml poly-L-lysine, and the whole medium was changed next day. After 10 days' culture, the cells were rinsed three times with culture medium and pre-shaken for 1 hour at 200 rpm to remove microglia. Then flasks were sealed and shaken at 250 rpm at 37°C for 15–18 hours to remove oligodendrocyte precursor cells. The medium with the detached cells was collected and first plated on tissue culture dishes (non PDL coated) for 30–60 minutes at 37°C with a gentle swirling of the dishes to eliminate contaminating dead cells and residual microglia. The nonadherent cells were collected, centrifuged and replated in DMEM containing 10% FBS.

For Nkx6.1 knockdown experiments, small interfering RNA for Nkx6.1 (siRNA-Nkx6.1) was prepared by GenePharma (Shanghai, China). The siRNA-Nkx6.1 sequence was 5′-GGA GAA GAC UUU CGA ACA ATT UUG UUC GAA AGU CUU CUC CTT. Purified astrocytes were transfected with siRNA-Nkx6.1 6 h before experiments using Lipofectamine 2000 according to the manufacturer's guidelines.

### RNA Preparation and Real-Time PCR

mRNA levels were assessed by real-time PCR using an Bio-Rad QX100 Droplet Digital PCR system (USA). cDNA was synthesized by Mol Neurobiol reverse transcription using oligo (dT) as the primer and proceeded to real-time PCR with gene-specific primers in the presence of SYBR Premix Ex Taq (DRR041A, Takara Biotechnology, Dalian, China). Quantification was performed by the comparative cycle threshold (Ct) method, using *β-actin* as the internal control. The following forward (F) and reverse (R) primers were used to amplify: *GFAP*-F: 5′-CCA CCA GTA ACA TGC AAG AAA CA-3′, *GFAP*-R: 5′-CAG TTG GCG GCG ATA GTC A-3′, *STAT3*-F: 5′-GCA TTC GGA AAG TAT TGT CGC-3′, *STAT3*-R: 5′-ATC GGC AGG TCA ATG GTA T-3′, *Olig2*-F: 5′-GCT GTG GAA ACA GTT TGG GT-3′, *Olig2*-R: 5′-AAG GGT GTT ACA CGG CAG AC-3′, *β-actin*-F: 5′-CGC ACC ACT GGC ATT GTC AT-3′, *β-actin*-R: 5′-TTC TCC TTG ATG TCA CGC AC-3′.

### Statistical Analysis

Statistical differences were determined by Student's t test for two-group comparison or by one-way ANOVA followed by Tukey's post hoc test for multiple comparisons. The accepted level of significance was P<0.05. Data in the text and figures are presented as mean±SEM.

## Results

### *Nkx6.1* is selectively expressed in ventral gray and white matter astrocytes

**Figure 1:**
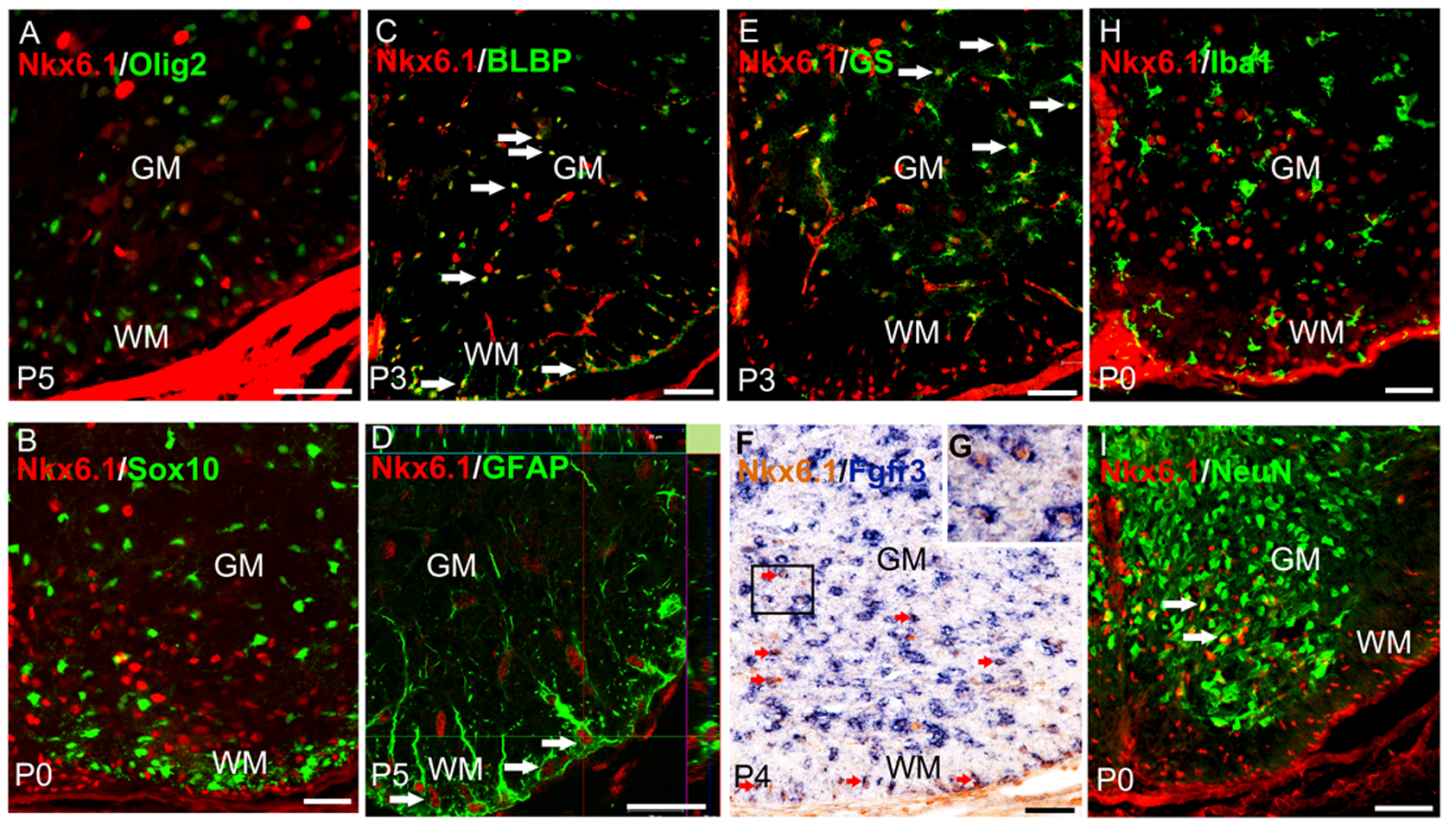
Nkx6.1 expression in ventral spinal cord astrocytes. **A-E**: Transverse sections from wild-type mice spinal cord at early postnatal stages were double immunostained with anti-Nkx6.1 in conjunction with anti-Olig2, anti-Sox10, anti-BLBP, anti-GFAP, or anti-GS. Double-positive cells are represented by arrows. Orthogonal reconstructions of confocal images at z-axis level are shown in side panels (along the right-hand edge and beneath). Note that all Nkx6.1+ cells in ventral white matter (WM) co-express GFAP. **F-G**: Spinal cord sections from P4 wild-type animal were subjected to Fgfr3 (blue) ISH followed by anti-Nkx6.1 (brown) immunostaining. Representative double positive cells in WM and gray matter (GM) are indicated by red arrows. The insets are the higher magnification of double positive cells. **H-I**: Transverse sections from wild-type mice spinal cord at P0 were double immunostained with anti-Nkx6.1 and anti-Iba1 or anti-NeuN antibodies. Arrows represent double-positive cells in GM. Scale bars 50 µm.

*Nkx6.1* is widely expressed by neural progenitor cells within the ventral neural tube throughout embryogenesis [11]–[14]. As the majority of ventral domains contribute to astrogliogenesis, it is likely that the *Nkx6.1* may regulate astrocyte development in the spinal cord. Immunostaining in postnatal mouse spinal cords revealed scattered Nkx6.1-expressing cells in the ventral spinal cords of early postnatal stages. Double immunostaining in postnatal spinal cord revealed that Nkx6.1+ cells did not co-express the oligodendrocytes markers Olig2 and Sox10, nor the microglia marker Iba1 (Figure 1A, B, H). Instead, they co-expressed the astrocytic progenitor markers BLBP and Fgfr3 [22]–[24] (Figure 1C, F, G). Detailed analysis revealed that Nkx6.1+ nuclei were localized in GS+ protoplasmic astrocytes and in a few NeuN+ neurons in the ventral gray matter (Figure 1E, I), and were intimately associated with GFAP+ fibrous astrocytic processes in the white matter (Figure 1D). These expression studies demonstrate that during gliogenesis stage, *Nkx6.1* is specifically expressed in the astrocyte lineage, but not in the oligodendrocyte lineage in the developing spinal cord.

### *Nkx6.1* is required for the migration of astrocyte progenitors

**Figure 2:**
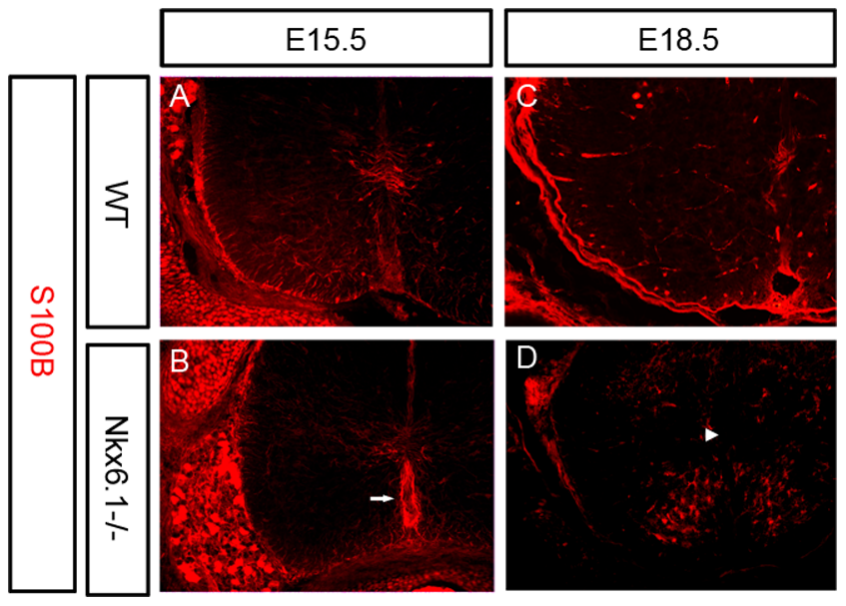
Abnormal S100B expression in *Nkx6.1* mutant spinal cord. Spinal cord sections from E15.5 (A-B) and E18.5 (C-D) wild-type (A, C) and *Nkx6.1−/−* (B, D) embryos were immunostained with anti-S100B. S100B expression was dramatically reduced in the ventricular zone (arrowhead), but increased in the floor plate (arrow) in the mutants. Scale bar: 100 µm.

To investigate the function of *Nkx6.1* in regulating astrogliogenesis in the ventral spinal cord, we first examined the effect of *Nkx6.1* null mutation on the expression of S100β, an early astroglial marker expressed in the VZ [25]. At embryonic day 15.5 (E15.5), S100β was predominantly expressed in the VZ (Fig 2A). In *Nkx6.1* mutant, ventricular expression of S100β was dramatically reduced, but its ectopic expression was detected within the floor plate (Fig 2B). At E18.5, ventricular S100β expression was still present in the wild-type tissue, but completely vanished in the mutant spinal cord (Fig 2C-D), suggesting that *Nkx6.1* mutation led to an abnormal fate specification of ventricular neural progenitor cells.

**Figure 3:**
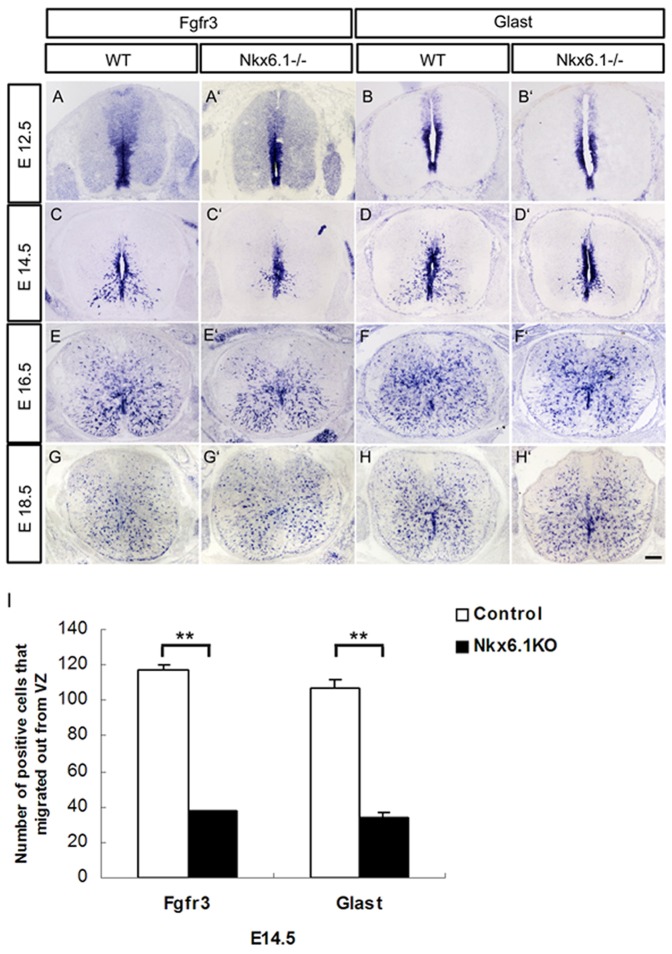
Delayed migration of astrocyte progenitors in *Nkx6.1* mutant spinal cord. **A-H′**: Transverse spinal cord sections from E12.5 (A-B′), E14.5 (C-D′), E16.5 (E-F′) and E18.5 (G-H′) wild-type and mutant embryos were subjected to ISH with riboprobes for *Fgfr3* and *Glast*. **I**: The number of Fgfr3+ or Glast+ cells migrated out from VZ in the control and Nkx6.1 mutant spinal cord at E14.5. Error bar, standard deviation (n = 3, **P<0.01). In *Nkx6.1* mutants, the early expression of *Fgfr3* and *Glast* in the ventral ventricular zone is not affected, but the migration of progenitor cells is significantly delayed at E14.5 although it is recovered at E18.5. Scale bar: 100 µm.

We next examined the expression of two other astrocytic progenitor markers, *Fgfr3* and *Glast* [22], [26], [27] in the ventral spinal parenchyma by RNA *in situ* hybridization (ISH). At E12.5, expression of *Fgfr3* and *Glast* was restricted to the ventricular zone of the wild-type and *Nkx6.1* mutant embryos (Figure 3A-B′). At E14.5, numerous *Fgfr3+* and *Glast+* astrocyte progenitors had migrated out into white matter in control embryos, but few were seen in the mutants (Figure 3C-D′, I). At later stages, the number of astrocyte progenitors in the white matter of *Nkx6.1* mutants increased rapidly (Figure 3E-F′). By E18.5, no apparent difference was found in the wild-type and mutant animals (Figure 3G-H′). These results suggested that *Nkx6.1* is required for the timingly generation and migration of astrocyte progenitors in the ventral spinal cord.

### Differentiation of fibrous astrocytes in ventral white matter is retarded in *Nkx6.1* mutants

**Figure 4:**
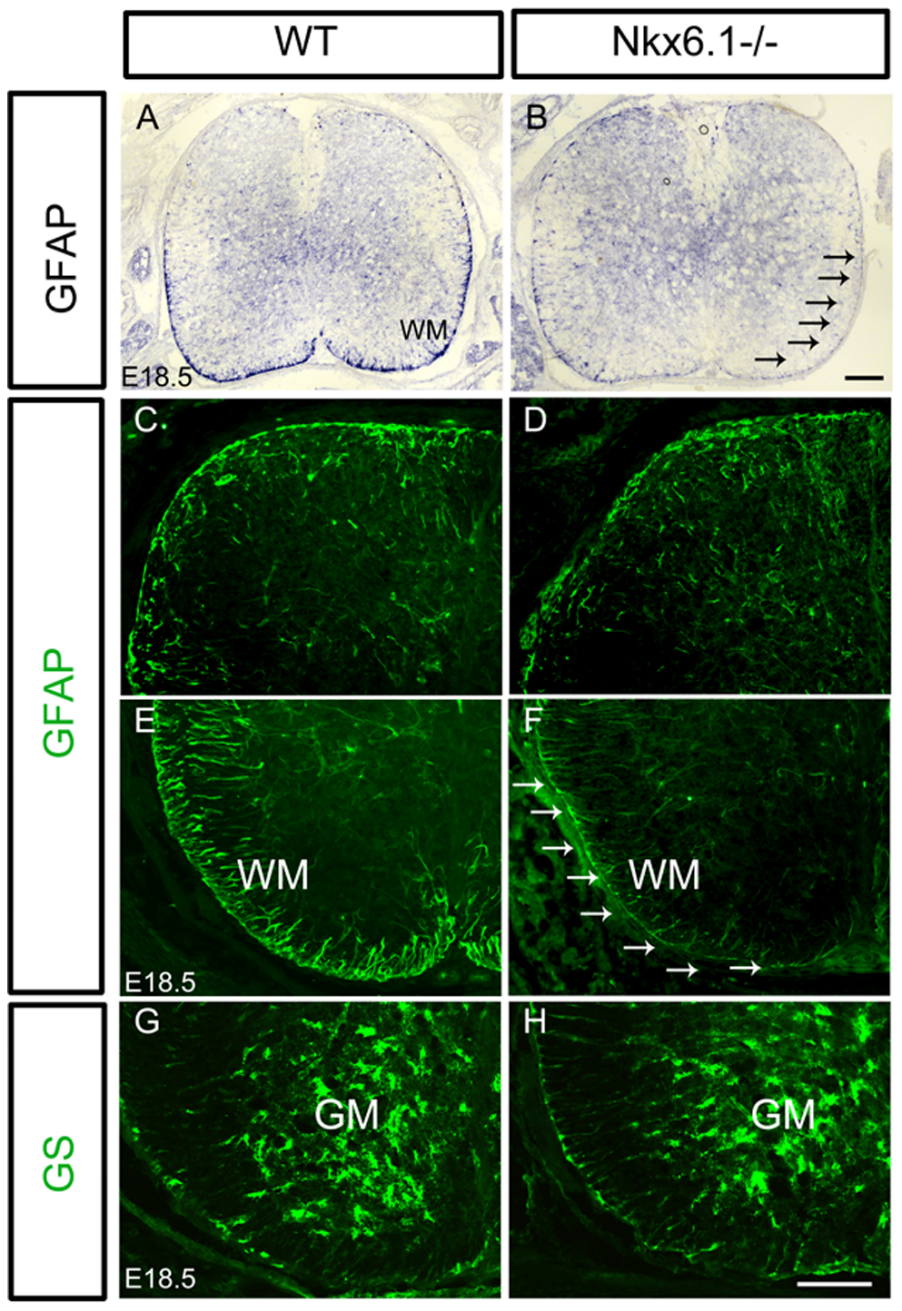
Defective differentiation of fibrous astrocytes in the ventral white matter of *Nkx6.1* mutant spinal cord. Spinal cord sections from E18.5 wild-type and *Nkx6.1* mutants are subjected to ISH with GFAP riboprobes (A, B), or immunostaining with anti-GFAP (C-F) or anti-GS (G, H). In *Nkx6.1* mutants, GFAP expression was reduced in the ventral, but not dorsal white matter. Strong GS signal was observed in the ventral gray matter of spinal cord, and no significant difference was detected between control and mutant tissues (G, H). Arrows indicate the reduced GFAP staining in the mutant tissues. Scale bars: 100 µm.

We next assessed whether the differentiation of astrocytes is also influenced by *Nkx6.1* mutation in the ventral spinal cord. GS and GFAP are lineage-specific markers that label protoplasmic in the gray matter and fibrous astrocytes in the white matter, respectively [28], [29]. At E18.5, GFAP is largely expressed by fibrous astrocytes in the ventral white matter in control animals (Figure 4A, E). However, a marked decrease of GFAP expression was observed in the ventral white matter of *Nkx6.1* mutant mice at both the mRNA (Figure 4B) and protein levels (Figure 4F). As expected, expression of GFAP in dorsal spinal cord was not affected by the mutation (Figure 4C, D). At the same stage, strong GS immunoreactivity was observed in the entire gray matter in both the wild-type and mutant spinal cords, and no significant difference was detected between these two genotypes (Fig 4G, H).

**Figure 5:**
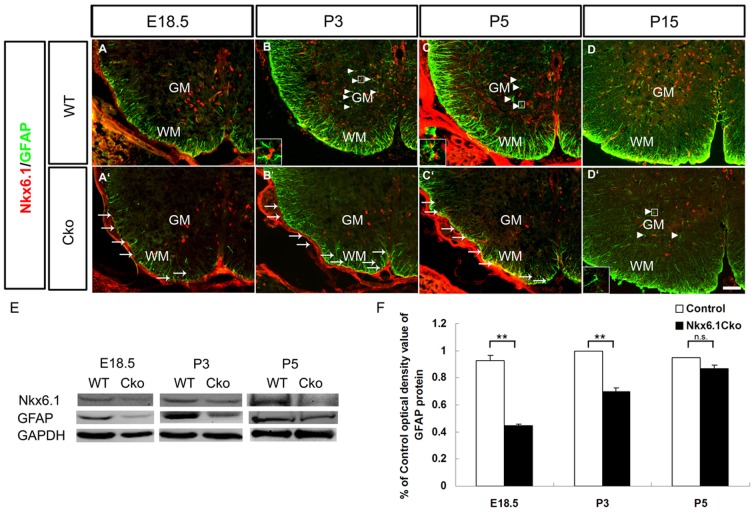
Delayed differentiation of fibrous astrocytes in *Nkx6.1* conditional mutants. **A-D′**: Spinal cord sections from E18.5 (A-A′), P3 (B–B′), P5 (C-C′) and P15 (D-D′) wild-type or *hGFAPCre;Nkx6.1^flox/flox^* (*Cko*) conditional mutant embryos were subjected to double immunolabeling with anti-Nkx6.1 and anti-GFAP. GFAP expression was dramatically reduced in the white matter of Cko spinal cord at E18.5 (A, A′), but mostly recovered at P15 (D, D′). GFAP+/Nkx6.1+ double positive cells are represented by arrows in the white matter, and arrowheads in the gray matter. The insets in B, C, and D′ are the higher magnification of cells in the boxes in gray matter. Scale bars: 50 µm. **E**: Western blotting of E18.5-P5 spinal tissues with antibodies against Nkx6.1, GFAP or GAPDH. **F**: Statistical analysis on the relative expression level of GFAP at E18.5, P3 and P5 stages with Student's t-test. Error bar, standard deviation (n = 3, **P<0.01, n.s. =  no significant).

Since *Nkx6.1* mutant animals die immediately after birth due to defective motor neuron development, it is not clear whether *Nkx6.1* function is required for postnatal astrocyte development. Thus, we generated the *hGFAPCre;Nkx6.1^flox/flox^* conditional mice (Cko) in which *Nkx6.1* expression was selectively ablated in the ventricular progenitor cells after neurogenesis [30] (Figure 5A′-D′). Consistent with the results observed in the *Nkx6.1* conventional knockouts, GFAP was significantly reduced in the ventral white matter of Cko mutants during embryogenesis (Figure 5A, A′). However, at postanatal stages, expression of GFAP in Cko mutants increased with time in the white matter (Figure 5B′, C′, D′), despite the persistently lower level of its expression than that in the wild-type animal at all stages examined (Figure 5B, C, D). Western blotting results revealed a similar reduction of GFAP protein expression in Cko mutant spinal cords from E18.5 up to postnatal P5 (Figure 5E, F). Together, these observations suggest that *Nkx6.1* ablation results in delayed expression of GFAP in the ventral spinal cord, indicating that *Nkx6.1* activity is vital for the timely differentiation of GFAP+ astrocytes in this region.

### *Nkx6.1* knockdown suppress astrocyte differentiation in culture

**Figure 6:**
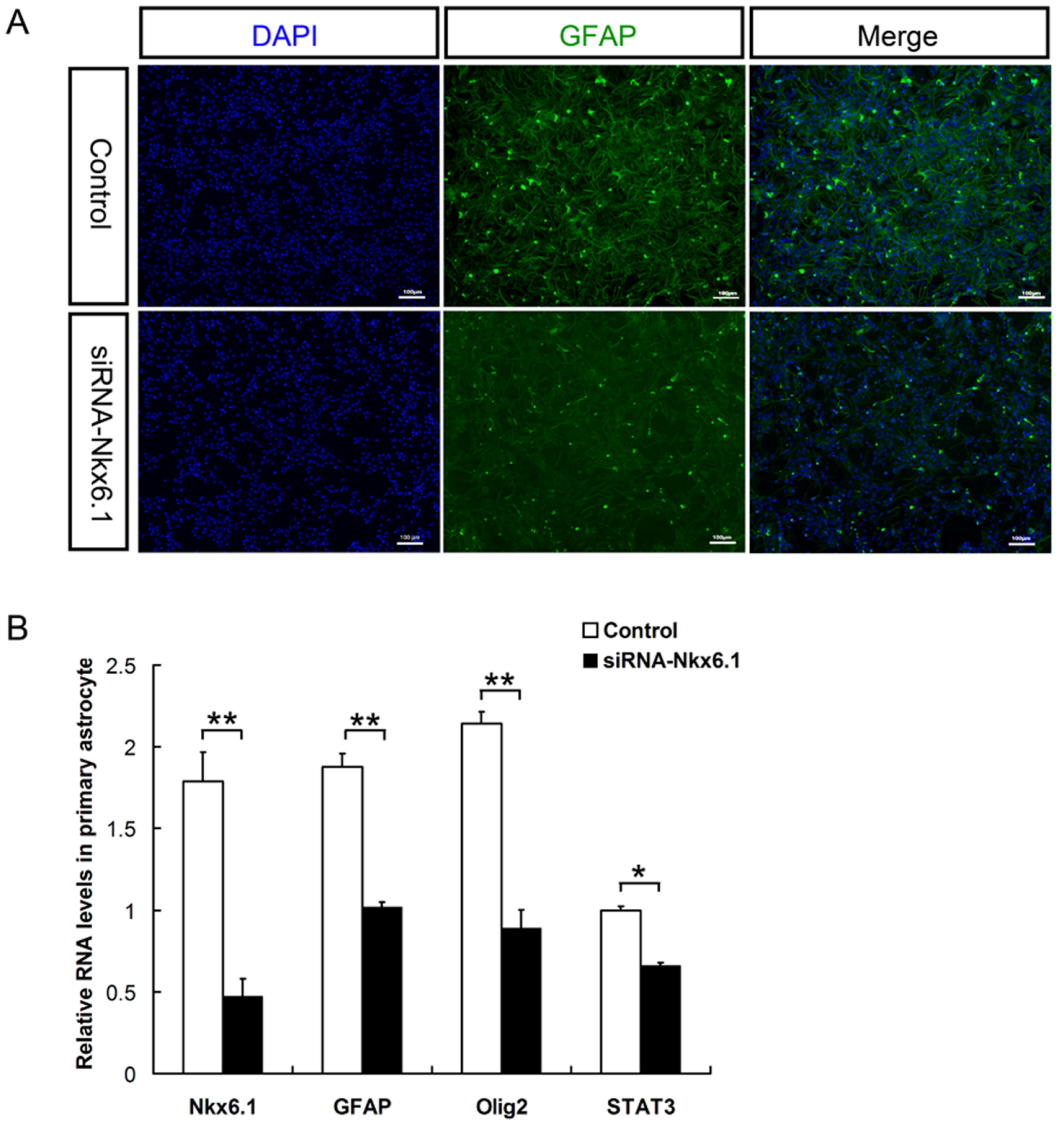
Effects of Nkx6.1 knockdown on astrocyte differentiation. **A**: Astrocytes were transfected with an siRNA-actin (Control) or siRNA-Nkx6.1 for 6 hours and immunostained with anti-GFAP antibody. Scale bar 100 µm. **B**: Realtime-PCR analysis of the mRNA levels of *Nkx6.1*, *GFAP*, *Olig2* and *STAT3* in astrocytes after transfection with Control or siRNA-Nkx6.1. The fluorescent output signal of each gene was measured and normalized to the corresponding control. (n = 3. *P<0.05. **P<0.01).

To address the cell-autonomous effects of *Nkx6.1* deficiency in astroglia lineage development, cultured astrocytes were treated with siRNA to suppress *Nkx6.1* expression. Consistent with the *in vivo* data, the treatment significantly reduced *GFAP* expression in primary astrocyte culture (Figure 6A), suggesting an autonomous role for Nkx6.1 in the regulation of astrocyte differentiation. Meanwhile, the expression of *Olig2* and *STAT3* was also reduced in the astrocytes treated with siRNA-Nkx6.1 (Figure 6B). Since *STAT3* is involved in astrocyte differentiation [31], it has raised the possibility that *Nkx6.1* may regulate astrocyte differentiation through the JaK/STAT signaling pathway.

### *Nkx6.1* is required for the generation of VA2 white matter astrocytes

**Figure 7:**
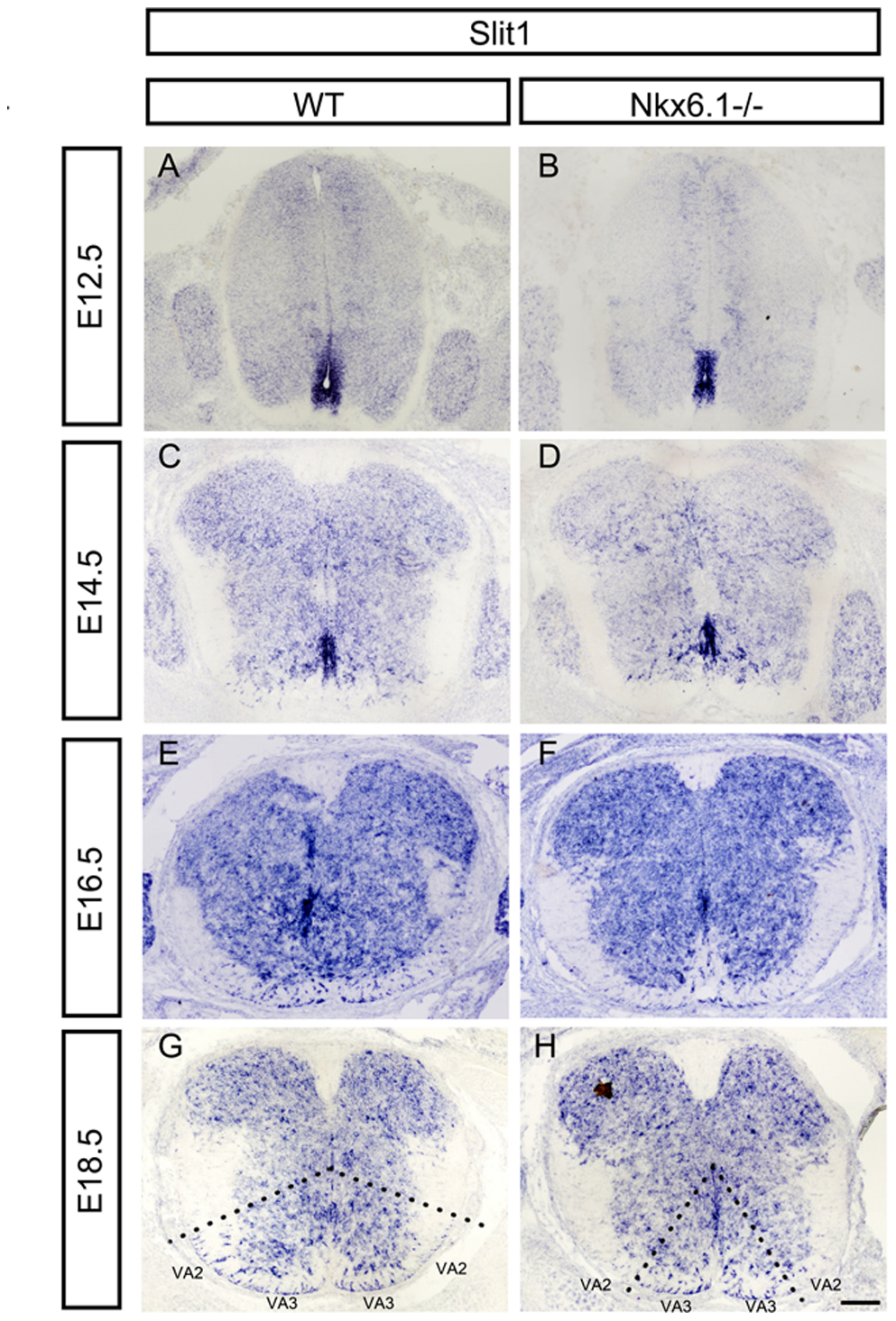
Abnormal Slit1 expression in spinal cords at embryo stages of Nkx6.1 mutant. Spinal cord sections from E12.5 to E18.5 embryos were subjected to ISH with *Slit1* riboprobe in WT and *Nkx6.1−/−* mice. *Slit1* expression in the ventral white matter was significantly reduced at E16.5. At E18.5, *Slit1* expression was observed in the ventral astrocyte subtypes 2 and 3 (VA2 and VA3) in wild-type embryos, but only in VA3 astrocytes in Nkx6.1 mutants (outlined by dashed lines in G- H). Scale bars: 100 µm.

It has been shown that there are at least three subtypes of white matter astrocytes in the ventral spinal cord, ventral astrocyte subtypes 1, 2 and 3 (VA1, VA2 and VA3) that are produced from p1, p2 and p3 progenitor domains, respectively [15]. It appears that *Nkx6.1* is sufficient for the emergence of *Slit1+* VA2 and VA3 astrocytes; however, it is not clear if *Nkx6.1* is required for VA2/3 astrocytes. Thus, we examined the *Slit1* expression in the spinal cord of Nkx6.1KO mice at various embryonic stages. Prior to gliogenic stages, *Slit1* was only expressed in ventricular neural progenitor cells (Figure 7A, B). At E14.5, many Slit1+ cells had migrated out into the white matter from p3 domain in the wild-type embryo; however, fewer *Slit1+* cells were detected in the ventral white matter in the mutants (Figure 7C-F). By E18.5, *Slit1* expression was observed in both VA2 and VA3 astrocytes in wild-type spinal cord, but only in VA3 astrocytes in *Nkx6.1* mutants (Figure 7G, H). These observations suggested that *Nkx6.1* is essential for the development of VA2 white matter astrocytes.

## Discussion

The *Nkx6.1* homeobox gene is expressed in the ventral neural progenitor cells that give rise to motor (MN), V2, and V3 neurons [12], [13] and ventral oligodendrocytes [19], [30]. Mice carrying *Nkx6.1* null mutation exhibit a ventral-to-dorsal switch in the identity of progenitor cells with a ventral expansion of V1 interneurons at the expense of MN and V2 neurons [16]. During gliogenesis, it causes a significant delay in OPC specification and differentiation [32], [7]. In this study, we found that *Nkx6.1* also plays a vital role in the development of astrocyte lineage in the spinal cord. *Nkx6.1* is continuously expressed in astrocyte precursor cells after they migrate from the VZ into the parenchyma (Figure 1). Similarly, S100B is also expressed by astroglial progenitor cells in the VZ and immature astrocytes that are dispersed within the mantle zone (Fig 2) [33], [34]. Disruption of *Nkx6.1* lead to a loss or severe reduction of S100B expression in the VZ at E15.5 and E18.5 stages (Figure 2). The lack of S100B expression in the mutants strongly suggests that *Nkx6.1* plays an essential role in the fate specification or lineage maintenance of astrocyte precursors in the ventral VZ. Consistently, we noticed that the expression of VA2 *Slit1* was lost from E16.5 ventral spinal cord in *Nkx6.1* null mice. This finding also indicated that *Nkx6.1* is required for the development of VA2 but not VA3 white matter astrocytes (Figure 7). It has been proposed that co-expression of Pax6 and Nkx6.1 specifies a VA2 phenotype (Reelin+, Slit1+) [15]. The normal *Reelin* expression in Nkx6.1 mutant spinal cord (Figure S1) suggests that VA2 astrocytes may acquire the VA1 phenotype (Reelin+/Slit1-), consistent with the finding that expression of Nkx2.2 and Pax6 in the VZ were not affected in the Nkx6.1 mutant [16].

Intriguingly, ectopic expression of S100B was observed in the floor plate of E15.5 spinal cords (Figure 2B), which may be caused by the abnormal migration of mis-specified ventricular cells. Concommitantly, migration of Glast+ and Fgfr3+ astrocyte progenitors from the ventral ventricular zone into the ventral parenchyma was also affected and delayed (Figure 3). Expression of mature astrocyte marker GFAP was also retarded in the ventral white matter (Figure 4). Moreover, the processes of GFAP+ fibrous astrocytes in the white matter become irregular (Figure 3, 4). In support of this notion, siRNA-Nkx6.1 treatment reduces *GFAP* expression in primary astrocyte culture in vitro (Figure 6), suggesting an autonomous role for Nkx6.1 in the regulation of astrocyte differentiation. Collectively, these observations demonstrate that *Nkx6.1* function is required for the normal differentiation and morphogenesis of ventral fibrous astrocytes.

The mechanisms underlying the retarded differention and maturation of astrocyte progenitor cells are currently unknown. Previous studies demonstrated that *Olig2* function is important for white matter astrocyte formation in the spinal cord at postnatal stages [16]. We found that *Nkx6.1* mutation leads to reduced expression of *Olig2* in the spinal cord at both embryonic [32] and early postnatal stages (Figure S2), it is plausible that the defective astrocyte specification and differentiation in *Nkx6.1* mutants may be caused by reduction in *Olig2* expression. Consistently, inhibition of *Olig2* expression was also observed in primary astrocytes when Nkx6.1 expression is knocked down (Figure 6B). Moreover, the expression of *STAT3* was also reduced in the astrocytes treated with siRNA-Nkx6.1 (Figure 6B). It raised the possibility that *Nkx6.1* regulated astrocyte differentiation through the JaK/STAT signaling pathway, given that STAT3 is required for astrocyte differentiation [31].

## Supporting Information

Figure S1 **Normal** ***Reelin*** **expression in spinal cords at embryo stages of Nkx6.1 mutant.** Spinal cord sections from E12.5, E14.5, E16.5 and E18.5 were subjected to ISH with *Reelin* riboprobe in WT and Nkx6.1−/− mice. *Reelin* was similarly expressed in WT and Nkx6.1−/− mice at all stages (**A-H**). **I,J**: The insets are the higher magnification of ventral astrocyte subtypes 2 (VA2) of WT and *Nkx6.1−/−* spinal cord at E18.5.Scale bars: 100 µm.
(TIF)

Figure S2 **Reduced Olig2 expression in postnatal** ***Nkx6.1*** **conditional mutant spinal cord.** **A-H**: Transverse spinal cord sections from E18.5 (A, B), P0 (C, D) P5 (E, F) and P10 (G, H) wild-type and *Nkx6.1 Cko* embryos were subjected to immunostaining with anti-Olig2. Arrows indicate the white matter region of the spinal cord. **I**: Western immunoblotting of E18.5- P10 spinal tissues with antibody against Olig2 or GAPDH. Scale bars: 50 µm. **J**: Statistical analysis on the relative expression level of Olig2 at E18.5, P3, P5 and P10 stages with Student's t-test. Error bar, standard deviation (n = 3, **P<0.01, n.s. =  no significant).
(TIF)
