# Supplementary figures and images for: Control of Astrocyte Progenitor Specification, Migration and Maturation by Nkx6.1 Homeodomain Transcription Factor
Source: PLoS One. 2014 Oct 6;9(10):e109171. doi: 10.1371/journal.pone.0109171 (PMC4186865; doi:10.1371/journal.pone.0109171)

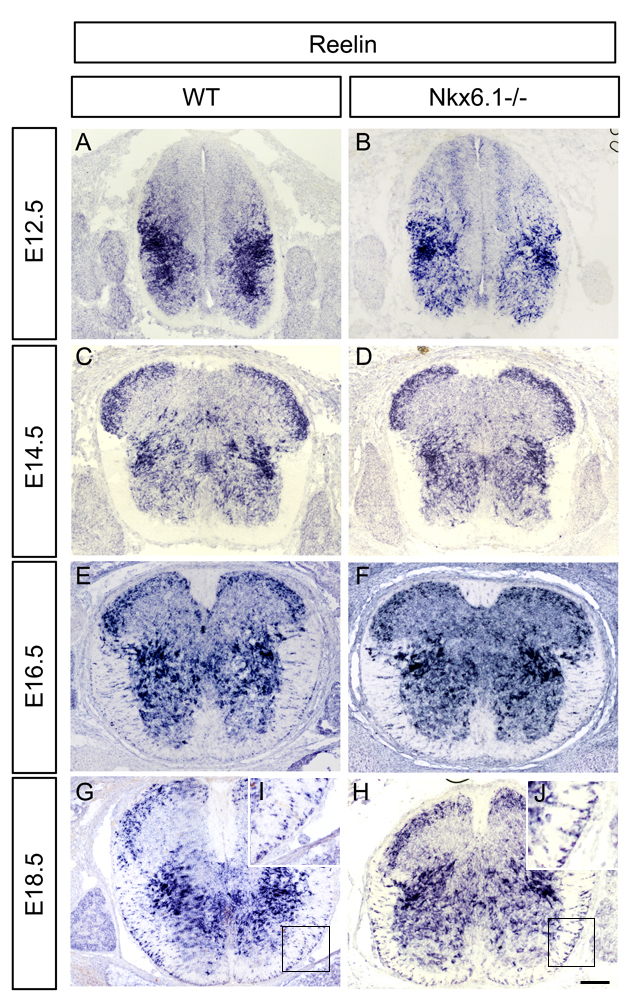

Supplement: Figure S1 — Normal Reelin expression in spinal cords at embryo stages of Nkx6.1 mutant. Spinal cord sections from E12.5, E14.5, E16.5 and E18.5 were subjected to ISH with Reelin riboprobe in WT and Nkx6.1−/− mice. Reelin was similarly expressed in WT and Nkx6.1−/− mice at all stages (A-H). I,J: The insets are the higher magnification of ventral astrocyte subtypes 2 (VA2) of WT and Nkx6.1−/− spinal cord at E18.5.Scale bars: 100 µm. (TIF) [file pone.0109171.s001.tif]

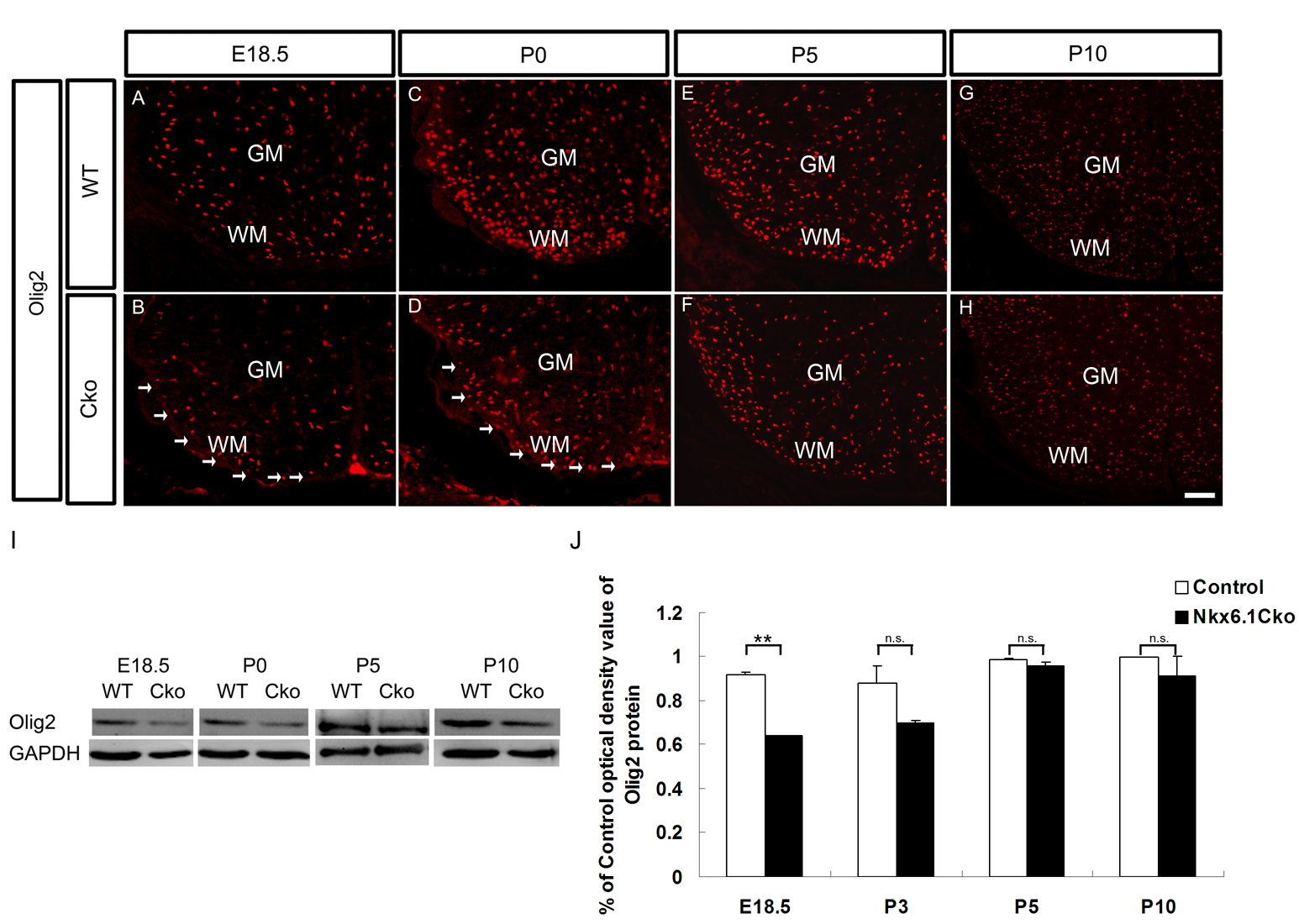

Supplement: Figure S2 — Reduced Olig2 expression in postnatal Nkx6.1 conditional mutant spinal cord. A-H: Transverse spinal cord sections from E18.5 (A, B), P0 (C, D) P5 (E, F) and P10 (G, H) wild-type and Nkx6.1 Cko embryos were subjected to immunostaining with anti-Olig2. Arrows indicate the white matter region of the spinal cord. I: Western immunoblotting of E18.5- P10 spinal tissues with antibody against Olig2 or GAPDH. Scale bars: 50 µm. J: Statistical analysis on the relative expression level of Olig2 at E18.5, P3, P5 and P10 stages with Student's t-test. Error bar, standard deviation (n = 3, **P<0.01, n.s. = no significant). (TIF) [file pone.0109171.s002.tif]
